# Multiple high-risk HPV infections probably associated with a higher risk of low-grade cytological abnormalities but not with high-grade intraepithelial lesions of the cervix

**DOI:** 10.1186/s12957-024-03360-2

**Published:** 2024-03-14

**Authors:** Tong Tong, Di Su, Qi Yang, Kun Yang, Yuqi Liu, Qun Wang, Tian Tian

**Affiliations:** Department of Reproductive Medicine, Department of Prenatal Diagnosis, The First Hospital of Jilin University, Changchun, Jilin China

**Keywords:** Human papillomavirus, Cervical cytology, Histopathology, Multiple infections

## Abstract

**Background:**

For women diagnosed with HR-HPV DNA positivity in community hospitals, the necessity of investigating the potential presence of multiple HR-HPV infections upon referral to tertiary medical institutions remains unclear.

**Methods:**

In our cohort, women tested positive for HR-HPV DNA during examinations in community hospitals, were subsequently referred to tertiary medical facilities, reevaluated HR-HPV genotype and categorized based on cytological and histopathological results. The risk of cytologic/histopathology abnormalities and ≧ high grade squamous intraepithelial lesion(HSIL) or Cervical Intraepithelial Neoplasia (CIN) 2 associated with individual genotypes and related multiple HPV infections are calculated.

**Results:**

A total of 1677 women aged between 21 and 77 were finally included in the present study. The cytology group included 1202 women and the histopathological group included 475 women with at least one HR-HPV infection of any genotype. We only observed a higher risk of low grade cytological abnormalities in women with multiple infections than those in corresponding single infections (for all population with an OR of 1.85[1.39–2.46]; *p* < 0.05). However, this phenomenon was not observed in histopathology abnormalities (CIN1). The risk of developing of ≥ HSIL/CIN2 in women who were infected with multiple HR-HPV also showed a similar profile to those with a single HR-HPV genotype.

**Conclusion:**

Multiple HR-HPV infections is only associated with a higher associated risk of low grade cytological abnormalities. There is no evidence of clinical benefit to identify the possible presence of multiple HR-HPV infection frequently in a short period of time for women with HR-HPV-DNA positive.

## Background

Cervical cancer is a worldwide concern because it is the fourth most common cancer among women worldwide; Out of more than 100 human papillomavirus (HPV) genotypes, 13–15 have been identified as high-risk HPV (HR-HPV) types, many of these infections are associated with only mild cellular morphological abnormalities, rarely progressing to cervical precancerous lesions, and only a small number of precancerous lesions actually develop into cancer [1]. Hybrid Capture 2 (QIAGEN) is one of the most common commercially available molecular tests for HR-HPV DNA detection, however, could not distinguish whether tumor-associated HPV DNA was indeed due to a single infections alone, or to multiple infections. The issue of multiple HR-HPV infections has recently aroused interest because it is common, with 28–50% of all HPV positive women having multiple infections [2–6].

However, the existence of detrimental effects of multiple HR-HPV infections remains controversial. First, persistent infection with HR-HPV has been shown to be a major risk factor for cervical cancer [7]. However, there is no conclusive evidence as to whether a multiple infection reduces the clearance rate of HR-HPV and increases the duration of persistent infection as compared with a single infection [8];Some researchers claim that multiple HR-HPV infections led to a prolonged infection duration [9], increase the incidence of high-grade squamous intraepithelial lesions (HSIL) and invasive cervical cancer (ICC) [2, 6, 10–15]. In contrast, there is also evidence that multiple HR-HPV infections were associated with a lower risk of high-grade cervical epitheliomatosis as compared to single infection [16–20]. Others have concluded that the effect of single and multiple HR-HPV infections on the severity of cervical tumors was comparable [21–23].

Confronted with these inconsistent results, clinical evaluations of multiple HPV infections have to be interpreted with caution. Community hospitals serve as the primary point of contact for women undergoing HR-HPV screening, playing a pivotal role in cervical cancer prevention and early detection. However, due to technological constraints, often limited to performing HR-HPV DNA assay, which lacks the capability to differentiate multiple infections. Therefore, we performed rescreening HR-HPV genotypes among those who were found HPV DNA positive in the community hospital, to evaluate whether multiple HR-HPV infections were associated with a higher risk of cervical diseases as compared with corresponding HR-HPV single infection.

## Materials and methods

### Study population and procedures

The study protocol was approved by the Ethics Committee of the First Hospital of Jilin University, in the province of Jilin, China (Ethics Committee Number 2020 − 653). Informed consents from patients were waived for this retrospective study. All methods were carried out in accordance with approved guidelines and the Declaration of Helsinki.

**Fig. 1:**
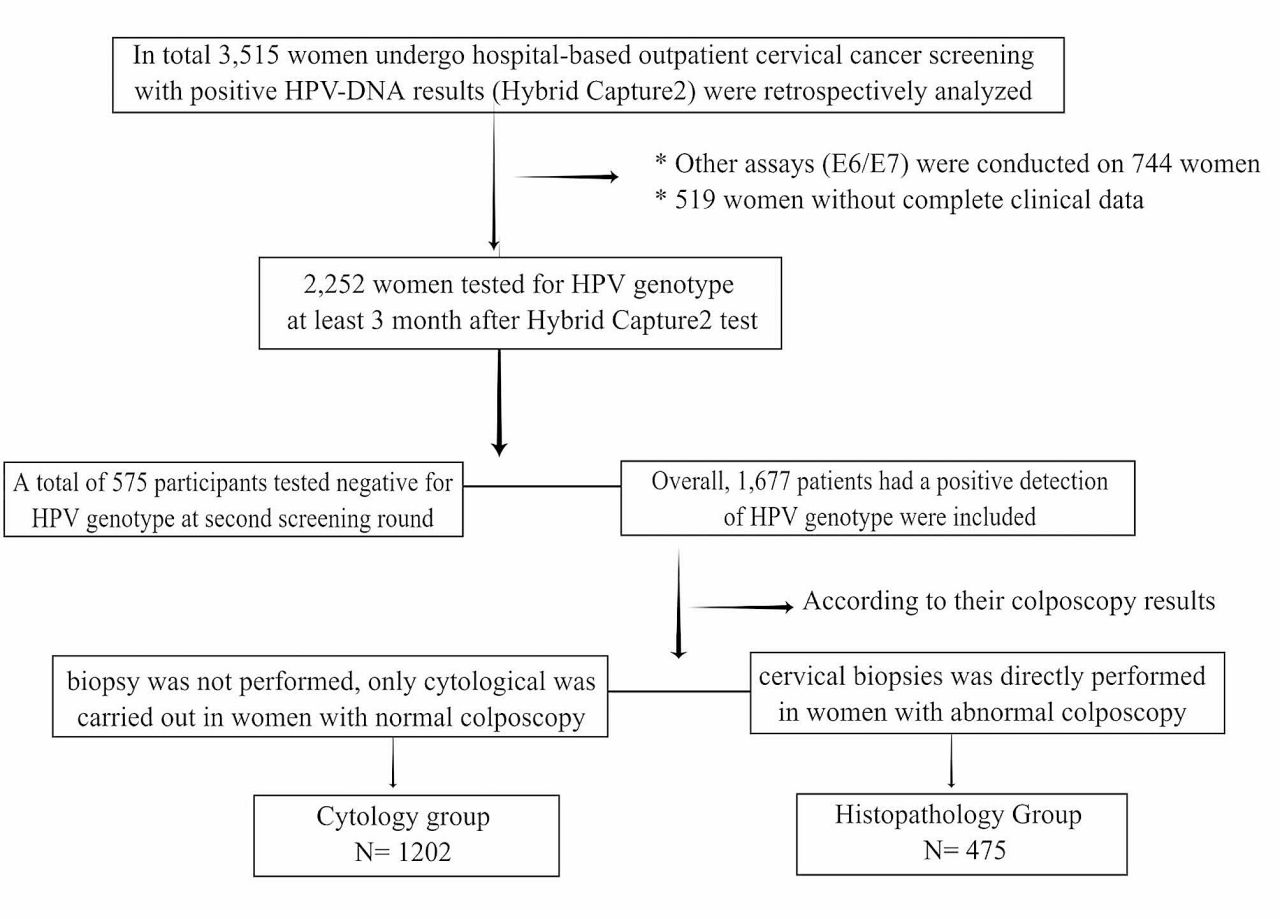
Flowchart for inclusion of patients

In this retrospective observational study, 3515 women were aged 20 years and older, without presence of definite cervical cancer, pregnancy and hysterectomy, actively screened for cervical cancer in community hospitals then visited the First Hospital of Jilin University outpatient clinic due to positive HPV-DNA results (Hybrid Capture2) from 2018 to 2019. The HPV-prevalence was re-assessed at the tertiary teaching hospital at least 3 months following the first cervical cancer screening in community hospitals. Of them, 1263 (35.9%) were excluded (744 were excluded because their re-assessment was performed by HPV E6 and E7 assays, 519 women without complete clinical data). The HPV-type distribution was re-assessed in 2,252 women: there were 575 women with HPV-positive at first screening and HPV-negative at the second screening round. HPV infections with single or multiple HPV types were considered if ≥ 1 h-HPV type were detected. In addition to HPV-type distribution examinations, according to colposcopy findings, TCT or cervical biopsies was performed. No biopsy was performed in women with normal colposcopy, only cytological examination was performed. Conversely, cervical biopsies was directly performed in women with abnormal colposcopy where lesions were visible. In the end, 1,677 subjects were enrolled with at least one HR-HPV infection of any genotype in the final analyses. The participants were separated into different groups according to different cervical cancer screening procedures. Of these, 1,202 women with both cytological and HR-HPV genotype positive results were included in the cytology group, 475 women with both histopathological and HR-HPV genotype positive results were included in the histopathology group. A patient inclusion flowchart is presented in the Fig. 1.

### HPV and cytologic/histopathology

Swab specimens were collected from each participant during a routine pelvic examination. The first specimen was collected from the cervix with a cytobrush, which was used for cervical TCT (Hologic, Marlborough, MA, USA). Using the Bethesda System 2001, a cytology result ≥ Atypical squamous cells of undetermined significance (ASC-US) was defined as “abnormal.” The others specimen was also collected from the cervix and stored at 4˚C in the standard media. The DNA of 15 h-HPV types (16, 18, 31, 33, 35, 39, 45, 51, 52, 53, 56, 58, 59, 66, and 68) isolation and purification were conducted according to the manufacturer’s instructions (Hybribio Limited, Chaozhou, China). All procedures were carried out in the standard clinical laboratory of the First Hospital of Jilin University. These cervical biopsies were taken when a suspicious premalignant was found during colposcopic examination, histopathology were classified into cervical intraepithelial neoplasia grade 1 (CIN1), CIN2, CIN3) and invasive cervical cancer (ICC) according to the Cervical Intraepithelial Neoplasia classification [24].

### Statistical analysis

Descriptive statistics were calculated for all available patient demographics and clinical characteristics as the baseline. HPV types were also analyzed according to the various species of viruses in the system, according to the characteristics of the HPV genome, especially the L1 nucleotide sequence, HPV types are divided into different species:α9 (types 16, 31, 33, 35, 52, and 58), α7 (types18, 39, 45, 59, 68, and 70), α6 (types53, 56, and 66) and α5(HPV51) [25]. The infection rate and multiple infection rates were calculated by the type and species of virus. The risk of cytological(ASCUS, LSIL) / histopathology (CIN1) abnormalities and the risk of ≥ HSIL/CIN2 in women with multiple HR-HPV infection were compared to women with correlate single HR-HPV infection were evaluated via unconditional logistic regression (age adjusted), with odds ratio (ORs) and 95% confidence intervals (CIs) calculated. Negative for intraepithelial lesions (cytological) or negative (histopathology) was used as a reference. SPSS 22.0 (SPSS Inc., Chicago, USA) was used for statistical analysis.

## Results

### HPV genotype distribution

In total 3,515 patients were screened and 1,677 women aged between 21 and 77 (median = 40) were finally included in the present study. There was no significant difference in age between the Cytology and Histopathological groups (40.25 ± 10.99 vs. 40.57 ± 10.31, *p* = 0.580). There was also no significant difference in age distribution between the two groups (*p* = 0.322). The age distribution was mainly concentrated on 30–39 years old (33.5 in Cytology group and 36.6 in Histopathological group). Similarly, there was no difference in the initial type-specific HR-HPV prevalence in this population of two groups (*p* = 0.139). The majority of our samples were single HPV infection, the proportion of single HPV infections in Cytology group was 75.0% and 71.1% in Histopathological group. (As shown in Table 1); HPV16 was the most common HR-HPV genotype (450, 26.8%), followed by HPV52 (290, 17.3%) and HPV58 (269, 16.0%). The proportion of women with multiple infections was 14.2% (238/1,677), HPV16 with other HR-HPV types accounted for the highest proportion of multiple infections, at 33.3% (146/438). (As seen in Table 2)

**Table 1:** Age, Cytology and HistopathologicalResults and HR-HPV Detection in the Study

Variable	Cytology group *N* = 1202	Histopathological group *N* = 475	*P*
Age (mean ± SD)	40.25 ± 10.99	40.57 ± 10.31	0.580
Age range (N, %)			0.322
21–29	223(18.6)	66(13.9)	
30–39	403(33.5)	174(36.6)	
40–49	316(26.3)	136(28.6)	
≧ 50	260(21.6)	99(20.8)	
Number of HR-HPV types(N, %)			0.139
1	901(75.0)	338(71.1)	
2	228(19.9)	109(22.9)	
3	49(4.1)	16(3.4)	
4	17(1.4)	10(2.1)	
5	7(0.6)	2(0.5)	

Data were expressed as mean ± standard deviation or number (percentage), when appropriate

Data were assessed with one-way ANONA or Chi-square test, when appropriate

SD: standard deviation; HR-HPV: high-risk HPV.

**Table 2:** HPV type identified in single and multiple infection stratified by cytology/Histopathological results in HPV positive women

Identified HPV type	Type of infection		Cytology		Total	Histopathological			Total
		NILM	ASC-US + LSIL	HSIL		Negative	CIN1	CIN ≧ 2	
HPV 16	All	196	56	47	299	58	47	46	151
	Single	139	27	34	200	42	30	32	104
	Multi	57	29	13	99	16	17	14	47
α5	All	81	32	3	116	16	26	3	45
	Single	40	8	1	49	8	13	1	22
	Multi	41	24	2	67	8	13	2	23
α6	All	184	98	3	285	46	46	10	102
	Single	98	37	0	135	19	13	3	35
	Multi	86	61	3	150	27	33	7	67
α7	All	260	72	11	343	61	46	17	124
	Single	145	25	7	177	28	24	4	56
	Multi	115	47	4	166	33	22	13	68
α9	All	617	164	86	867	151	127	106	384
	Single	411	74	55	540	95	66	68	229
	Multi	206	90	31	327	56	61	38	155
Total without 16	All	692	176	35	903	146	125	53	324
	Single	555	117	29	701	108	86	40	234
	Multi	137	59	6	202	38	39	13	90

NILM: Negative for intraepithelial lesion or malignancy; ASC-US: Atypical squamous cells of undetermined significance; LSIL: Low grade squamous intraepithelial lesion; ASC-H: Atypical squamous cells, rule out HSIL; HSIL: High grade squamous intraepithelial lesion; HR-HPV: high-risk HPV; Cervical Intraepithelial Neoplasia: CIN.

α5: HPV51; α6:HPV53,56,66; α7:HPV18,39,45,59,68; α9:HPV16,31,33,35,52,58.

The total outnumbers the different combinations identified because different types are counted more than once between rows.

### Multiple HR-HPV infections associated with higher risk of low-grade cytological abnormalities

**Fig. 2:**
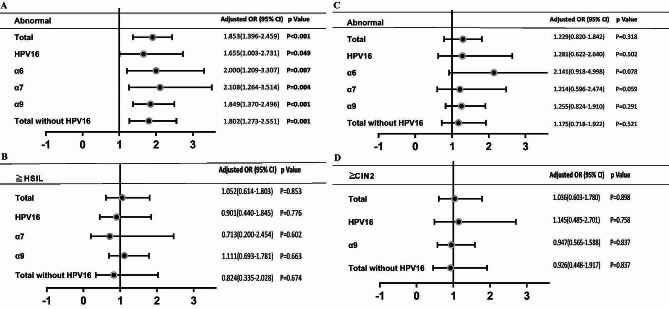
Cytology/Histopathology result stratified by type of HPV infection (multiple VS single), cytology abnormal results (ASCUS, LSIL) (**A**) and in the subgroup histopathology ≥HSIL results (**B**), histopathology abnormal results (CIN1) (**C**) and in the subgroup histopathology ≥CIN2 results (**D**) in the population studied α6:HPV53,56,66; α7:HPV18,39,45,59,68; α9:HPV16,31,33,35,52,58; > 4 women affected were included in the analyses; All Odds Ratios in comparison to NILM group and adjusted for age

Low grade cytology abnormal (ASCUS, LSIL) in women with multiple HR-HPV infections were significant higher than single infection when compared to NILM group, with an OR of 1.85[95%-CI: 1.39–2.46] (all population), 1.66[1.03–2.73] (HPV16-positive population), 2.00[1.21–3.31](α6-positive population), 2.11[1.26–3.51] (α7-positive population) and 1.85[1.37–2.50](α9-positive population) (Fig. 2A).

However, the risk of developing of ≧ HSIL in women who were infected with a single HR-HPV genotype showed a similar profile to multiple HR-HPV infections. Analyses were also repeated after excluding women with HPV16-related multiple HR-HPV infection. And we found that the corresponding results were not materially altered (Fig. 2B).

### Multiple HR-HPV infections did not increase the risk of histopathological abnormalities

In contrast, in histopathology abnormalities did not show a similar trend. Multiple HR-HPV infections demonstrated no obvious differences in low grade histopathology abnormalities (CIN1) than single infection when compared to normal group and adjusted for age, with an OR of 1.23[95%-CI: 0.82–1.84] (all population), 1.28[0.62–2.64](HPV16-positive population), 2.14[0.92–4.99](α6-positive population), 1.21[0.60–2.47] (α7-positive population) and 1.25[0.82–1.91](α9-positive population) (Fig. 2B). The risk of developing of ≧ CIN2 between women who were infected with a single HR-HPV genotype showed a similar profile to multiple HR-HPV infections (Fig. 2C, D).

Upon case counts restriction, number of ≧ HSIL in α6 positive population and ≧ CIN2 in α6/α7 positive population were less than 5, were excluded for statistics. Since the HPV16 contributes as much as 24.9% (299/1,202) of the HR-HPV infections, analyses were repeated after excluding women with HPV16-related multiple HR-HPV infection and did not yield very different results (Fig. 2 ).

## Discussion

The present study was conducted on women seeking cervical cancer screening in tertiary hospitals who diagnosed with HR-HPVDNA positivity in community hospitals. A positive HPV DNA test does not necessarily mean that there is an HPV infection, since it is possible for the virus to deposition on the skin surface [26], and most of HR-HPVs clear spontaneously within 6 to 9 months [27, 28]. As in the current study, 575 (25.5%) women with HR-HPV DNA positive at first screening turned negative at the second screening round after a period of 3 months. We assessed the risk of cytological/histopathology abnormalities and developing of ≧ HSIL/CIN2 associated with specific HR-HPV genotypes and multiple infections. In our study we found women co-infected with HR-HPVs only seems to have a higher associated risk of low grade cytological abnormalities, was no association with the developing of ≧ HSIL/CIN2.

Regarding the relationship between multiple HPV infections and cervical lesion, inconsistent results have been reported. There are several points of view, on one hand several investigators reported that a single histological cervical lesion is usually caused by a single genotype, and each HR-HPV genotype independently leads to cytological changes and contributes towards the risk of cancer, even if there are multiple HPV genotypes identified in cytological specimens [29–31], The ability to the developing of cervical lesion can be considered comparable between multiple and single HR-HPV infections [21–23]; On the other hand, some investigators suggested a synergistic interaction between different HR-HPV genotypes, multiple infections with HR-HPV exhibited even higher-grade cervical lesion [2, 6, 10, 11, 13–15]; Still others who do not identify as such a synergistic interaction, as in HPV genotypes with different risk categories is similar to the highest risk category, they more tend to attribute the increased risk of cytological abnormalities caused by multiple infections to the addition of individual carcinogenic HPV genotypes rather than synergistic interactions between different genotypes [12, 22]. Finally, other researchers believed that such a multiple infection attenuated the oncogenic effects of each HR-HPV individual, the risk of HSIL or ≧ CIN3 was much higher in single HR-HPV infections than those with multiple HR-HPV infections [17–20, 32]. A large-scale cervical cancer screening in other areas of China, Wu et al. [17] showed that compared to women with single HPV16 infection, lower risk of ≧ CIN3 in individuals with HPV16 multiple infections was found (with an OR of 0.63 [95%-CI: 0.493–0.822]). Jing et al. [32] conducted a study on 3226 women who were screened for cervical cancer, and found that compared with Low Risk HPV, the risk of HSIL was much higher in single HR-HPV infections (6.32 to 10.49 times) than those with HR-HPV multiple infections (4.99 times).

Overall, there is no consensus on the association of multiple infections with occurrence or progression of cervical cancer, this could be associated with multiple factors such as patients were recruited from different sources or geographical distribution: Patient recruitment was varied across the studies, for example, participants in these articles tend to be taken from women who were screened for cervical lesions from general population [12, 13, 17, 21, 32] and those from tertiary hospitals [2], with abnormal pap test or an abnormal colposcopy [11, 20, 23], with abnormal cervical cytology / histopathology [6, 10, 18, 19, 22], pathological specimens where HPV expression was analyzed retrospectively [15]. Different regions may also have some impact on the results, researchers in Portugal [2], the United States [12], and Brazil [13] believe that multiple infections lead to a higher risk of high-grade cervical epitheliomatosis compared to single infections, while other studies conducted in China [17, 32], and Mexico [21] suggest that multiple infections do not increase the risk of high-grade cervical epithelial neoplasia, and actually tend to be a protective factor instead.

The risk of cervical epithelial lesions often completely differs from that of different HR-HPV, for HPV16 single infections, the two-year cumulative risk of CIN2 and CIN3 + was 50.6% and 39.1% respectively; For other HR-HPV types, the risk for CIN2 ranged from 4.7 to 29.5%, and the risk for CIN3 + ranged from 0.0–14.8% [22]. HPV16 contributes as much as 33.3% (146/438) of the multiple infections in our study. Previous studies have shown that although the risk of cytological abnormalities in all types of multiple infections is three times higher than that of single infections, but there was no significant difference between multiple and single infections in the HPV16 [21].

As the focus gradually shifts towards primary and secondary prevention, which includes vaccines and HPV screening, there is a need to further evaluate the relationship between specific HR-HPV genotypes or species and cervical cytological abnormalities, precancerous lesions, and cervical cancer in China. The advantage of the present study lies in the large sample of HR-HPV genotypic data and cytological/histopathology results, carried out in a solitary and experienced laboratory. In the meantime, we also note some limitations in our study: Firstly, our study was cross-sectional in nature, we cannot evaluate whether the multiple infections were acquired simultaneously or sequentially. Secondly, follow-up data was not available for cytological abnormalities, the prevalence of cervical precancerous lesions was underestimated because CIN3 was common in women with ASC-US and LSIL cytology [6, 22]; Another limitation of the current study is the lack of referral of women with negative results to colposcopy and the fact that biopsy was only performed in abnormal colposcopy cases might have resulted in bias. As in 2112 women with abnormal cytology, 56% were found to have CIN2/CIN3 detected during colposcopy with targeted biopsy [33]. Finally, our study is based on a cross-sectional study conducted in a tertiary hospital, participants in this trial are mostly outpatients, it is necessary to take into account the issue of patient compliance: As women with HR-HPV infections, even positive in HPV 16 or 18, a subset of participants were consented only for TCT. Furthermore, some patients do not simply seek cervical cancer screening for the sake of screening alone, as they may also have other symptoms such as irregular vaginal bleeding and abnormal secretions. As mentioned above, may amplified selection bias and confounding bias in our study. Therefore, our conclusions must be regarded with caution.

## Conclusions

In summary, this study describes the incidence of cytological/histopathology abnormalities in Chinese women who were HPV-DNA positive in community hospitals and retested for HPV genotypes. We found multiple HR-HPV infections might be connected only with a higher associated risk of low grade cytological abnormalities, the risk of developing of ≧ HSIL and histopathology abnormalities both showed a similar profile to a single HR-HPV genotype infections. So for women with HR-HPV-DNA positive in the first screening, there is no evidence of clinical benefit to identify the possible presence of multiple HR-HPV infection frequently in a short period of time. Further prospective studies are needed to validate and determine the mechanisms of these multiple infections.

## Data Availability

The datasets used and/or analyzed during the current study are available from the corresponding author on reasonable request.

## Abbreviations

TCT: ThinPrep cytology test
NILM: Negative for intraepithelial lesion or malignancy
ASC-US: Atypical squamous cells of undetermined significance
LSIL: Low grade squamous intraepithelial lesion
ASC-H: Atypical squamous cells, rule out HSIL
HSIL: High grade squamous intraepithelial lesion
HR-HPV: High risk human papillomavirus
CIN: Cervical Intraepithelial Neoplasia
α5: HPV51
α6: HPV53,56,66
α7: HPV18,39,45,59,68
α9: HPV16,31,33,35,52,58
